# Carbamylated monomeric allergoids for sublingual immunotherapy in pediatric respiratory allergies

**DOI:** 10.5415/apallergy.0000000000000203

**Published:** 2025-06-02

**Authors:** Chang-Keun Kim, Enrico Compalati, Zak Callaway

**Affiliations:** 1 Asthma & Allergy Center, Inje University Sanggye Paik Hospital, Seoul, Korea; 2 Casa di Cura Villa Serena, GVM Care & Research, Genoa, Italy; 3 Science Division, Mahidol University International College, Nakhon Pathom, Thailand

**Keywords:** Allergen immunotherapy, allergic rhinitis, allergoid, asthma, children

## Abstract

Allergen immunotherapy (AIT) is an evidence-based therapy for allergic rhinitis and allergic asthma. AIT is largely recognized as the only causal treatment of allergic diseases that targets the underlying pathophysiology and may have a disease-modifying effect in addition to the antisymptomatic effect. Carbamylated monomeric allergoids (CMAs) are chemically modified allergens with reduced IgE-binding activity (reduced allergenicity) but full immunogenicity. The carbamylation process allows them to be much smaller than other modified allergens, making them ideal for sublingual immunotherapy (SLIT), and reduced allergenicity makes them safe and well tolerated. CMAs have several advantages over other SLIT products: smaller size for easier absorption through mucosa, greater resistance to proteolytic degradation, greater bioavailability, and reduced allergenicity with full immunogenicity. The tablet form allows for accurate dosing and compliance is easy to monitor. Safety is an especially important consideration when treating conditions in pediatric populations, as is patient compliance. This review focused on the efficacy, safety, and clinical application of monomeric allergoid SLIT for allergic disease in children and its suitability as an alternative to subcutaneous immunotherapy.

## 1. Introduction

Allergen immunotherapy (AIT) is an evidence-based therapy for both allergic rhinitis (AR) and allergic asthma (AA) [1, 2] and involves administering repeated doses of offending allergens to a person with allergic disease, to modulate the immune response with the eventual goal of reducing or eliminating adverse clinical responses to future allergen exposures [3]. AIT has been shown to reduce symptoms and medication use in patients with AR and asthma; in fact, it is the only therapy directly affecting the underlying pathophysiology by limiting or even stopping the occurrence of new sensitizations and clinical disease progression [4, 5]. AIT is recommended for children and adolescents, as well, given this age range is an ideal window due to the “plasticity” of the young immune system [6]. Although not fully elucidated, there are many possible mechanisms of action, with one being through its effect on T-helper 2 (Th2) responses, T-cells closely related to effector cells.

There is strong clinical, epidemiologic, anatomic, and pathophysiologic evidence that the upper and lower airways function as one unit [1, 7–9], so there is merit in treating AR and asthma as 2 parts of the same disease (ie, united airway disease) [10]. Most patients with AA have concurrent AR, and 10% to 40% of those with AR have AA [11]. Administering AIT to children with AR has been shown to potentially prevent AA onset [12], and pediatric patients with AA receiving treatment for AR have demonstrated improved AA control [13].

There are a number of administration routes for AIT, each with its advantages and disadvantages. Subcutaneous immunotherapy (SCIT) presents the risk of inducing local and systemic side effects as a result of allergen injection, and injections in general are not well taken by young patients. Studies monitoring large numbers (>10,000) of patients have shown a small but potential risk of adverse reactions, sometimes severe or life-threatening, mainly when some associated conditions coexist [14, 15]. New administration routes (eg, sublingual immunotherapy [SLIT]) have been developed and investigated as alternative options to the traditional and well-documented SCIT, with generally positive efficacy and safety findings in adults [16–18]. SLIT has the advantage of self-administration at home and appears safer than SCIT, although local oromucosal side effects appear quite frequently and may be bothersome to some patients, affecting treatment compliance. Other novel administration routes include intralymphatic immunotherapy (ILIT) and epicutaneous immunotherapy (EPIT). ILIT involves ultrasound-guided injections into the inguinal lymph nodes 1 month apart [19]. The major advantages of ILIT are its short duration and low allergen doses. EPIT developed from the knowledge that the skin has large numbers of antigen-presenting cells (APCs), which increase allergen presentation while preventing systemic allergen reactions [20]. Patches with absorbed allergens take the place of needles, providing better comfort and increased patient compliance [21, 22].

Carbamylated monomeric allergoids (CMAs) are chemically modified allergens with reduced IgE-binding activity (reduced allergenicity) but full immunogenicity. The carbamylation process allows them to be much smaller than other modified allergens, making them ideal for SLIT, and reduced allergenicity makes them safe and well tolerated. Safety is an especially important consideration when treating conditions in pediatric populations, as is patient compliance. Pharmacovigilance studies of CMAs have shown a very limited number of adverse drug reactions (ADRs). Due to their unique chemical structure, CMAs experience higher absorption rates and higher concentrations in the blood than other SLIT products. Having a short induction phase (ie, up-dosing phase) of 4 days is a definite advantage as patients receive maximum doses earlier, which may lead to earlier clinical benefits. SLIT with CMAs, therefore, warrants special attention in AIT.

This review focuses on the efficacy, safety, and clinical application of monomeric allergoid SLIT for allergic disease in children and its suitability as an alternative to SCIT.

## 2. Mechanisms of allergic disease

Allergic symptoms usually start in childhood after sensitization to common allergens such as house dust mites (HDM), animal dander, and tree pollen. Many immune cells, such as T-helper 2 (Th2) lymphocytes, eosinophils, mast cells, and basophils, contribute to allergic inflammation. Th2 cells produce interleukin (IL)-4, IL-5, IL-9, and IL-13, which then induce effector cells like eosinophils, mast cells, and basophils. Th2 cells also drive B-cells to produce allergen-specific IgE [23]. The Th2 profile found commonly in many atopic diseases may be because of increased differentiation and clonal expansion of Th2 cells or increased cell death of high IFN-γ-producing T-helper 1 (Th1) cells [24].

### 2.1. Allergy development

During initial exposure to an allergen (ie, allergic sensitization), epithelial cell-derived cytokines and chemokines may be released, which recruit dendritic cells (DCs) to the inflammation site. These epithelial-derived mediators promote the differentiation of immature DCs to proallergic type 2 DCs and activate tissue-resident group 2 innate lymphoid cells to release IL-5 and IL-13 [25]. Type 2 DCs then migrate toward the lymph nodes and promote differentiation of naive T-cells into Th2 cells, activated allergen-driven allergen-specific Th2 cells, and T follicular helper (Tfh) cells, which sustain allergic inflammation [26]. Tfh2 cells produce type 2 cytokines like IL-4, IL-5, and IL-13, along with Th2 and allergen-specific Th2 cells, and promote B-cell differentiation into IgE-producing plasmablasts and plasma cells. Secreted IgE binds to the surface of effector cells and, on subsequent exposure, cross-linking of IgE by allergens results in mast cell and basophil degranulation [27]. These immunologic events can lead to specific pathological conditions such as asthma, rhinitis, dermatitis, and less frequently systemic anaphylaxis.

Proper immune system maturity during the first years of life is essential in avoiding the development of AA. Abnormal regulatory T-cell (Treg) function and/or numbers have been identified as a main cause of AA, and defective Tregs have even been observed in umbilical cord blood in newborns genetically predisposed to allergy [28, 29]. Ninety percent of asthmatics are diagnosed by 6 years of age, which suggests that early-life events like atopic diseases and respiratory virus-induced wheezing illnesses exert a strong influence on AA development [28, 30]. It is therefore imperative to precisely diagnose and treat the root cause of allergic disorders at an early age. Early therapeutic interventions would be greatly aided by validated and reliable biomarkers that can be used for identifying those patients most likely to benefit from AIT.

## 3. AIT administration routes

SCIT was the first immunotherapy route introduced into clinical practice in the early 1900s and has been found to be highly effective for seasonal and perennial allergic diseases. However, SCIT has several limitations such as frequent hospital visits and required assistance of a medical professional, local reactions at the injection site (eg, swelling, redness, and pain), patient anxiety over needles, and rare instances of severe ADRs like anaphylaxis [31]. Reports of these ADRs from SCIT led to the development of SLIT in the 1980s, which showed promise as a safe and effective alternative option. SLIT delivers allergenic proteins in liquid preparations or tablet form, with or without a variable escalation phase (aka “up-dosing”). Liquid preparations are administered sublingually (ie, under the tongue), while the tablet is dissolved in the patient’s mouth in 1 or 2 minutes. SLIT can offer advantages in terms of patient acceptability and doctor’s handling if routine treatment programs are implemented to maintain compliance, narrow the range and decrease the severity of side effects, facilitate self-administration, and improve treatment of children afraid of needles [32]. Slight variations in SLIT products can affect patient compliance/adherence. A recent study of single-dose containers vs multiple-dose vials (both liquid preparations) vs tablet found patient compliance to be lowest in the multiple-dose vial group, with a significantly greater dropout rate compared with the other 2 products (*P* = 0.024) [33].

Compliance/adherence is a major consideration for AIT efficacy, and estimates vary widely (18–90%). Therefore, it is important that patients are properly assessed and educated (eg, treatment administration routes/schedules, benefits/risks, duration, and cost) [34] before embarking on a treatment course. Poor adherence occurs mainly due to side effects, inconvenience, perceived ineffectiveness, or forgetting to use [35].

## 4. Allergoids and CMAs

AIT safety became an issue due to reports of severe ADRs [36], which led to innovative efforts to produce safer AIT products by reducing allergenicity while maintaining immunogenicity. Allergoids are purified, modified allergens with an altered protein structure, resulting in weakened allergenicity and, therefore, improved safety. They were first developed in 1973 to reduce the side effects of SCIT by reducing the IgE-binding properties. Native allergens are chemically treated by polymerization with glutaraldehyde or formaldehyde as a cross-linking agent and result in high molecular weight molecules (200,000 to 20,000,000 Da) [32]. IgE-binding sites become inactive because of their altered chemical structure and impairment of conformational epitopes; therefore, overall, fewer IgE antibodies are bonded. Indeed, a 2024 study by Asllani et al. [37] found that the use of allergoids induced a lower risk of ADRs. However, immunogenicity and therapeutic effect remain the same because T-cell epitopes are preserved [38]. Due to the high molecular weight impairing mucosal uptake, these polymeric allergoids are not optimal for SLIT.

### 4.1. Carbamylated monomeric allergoids

**Figure 1.**
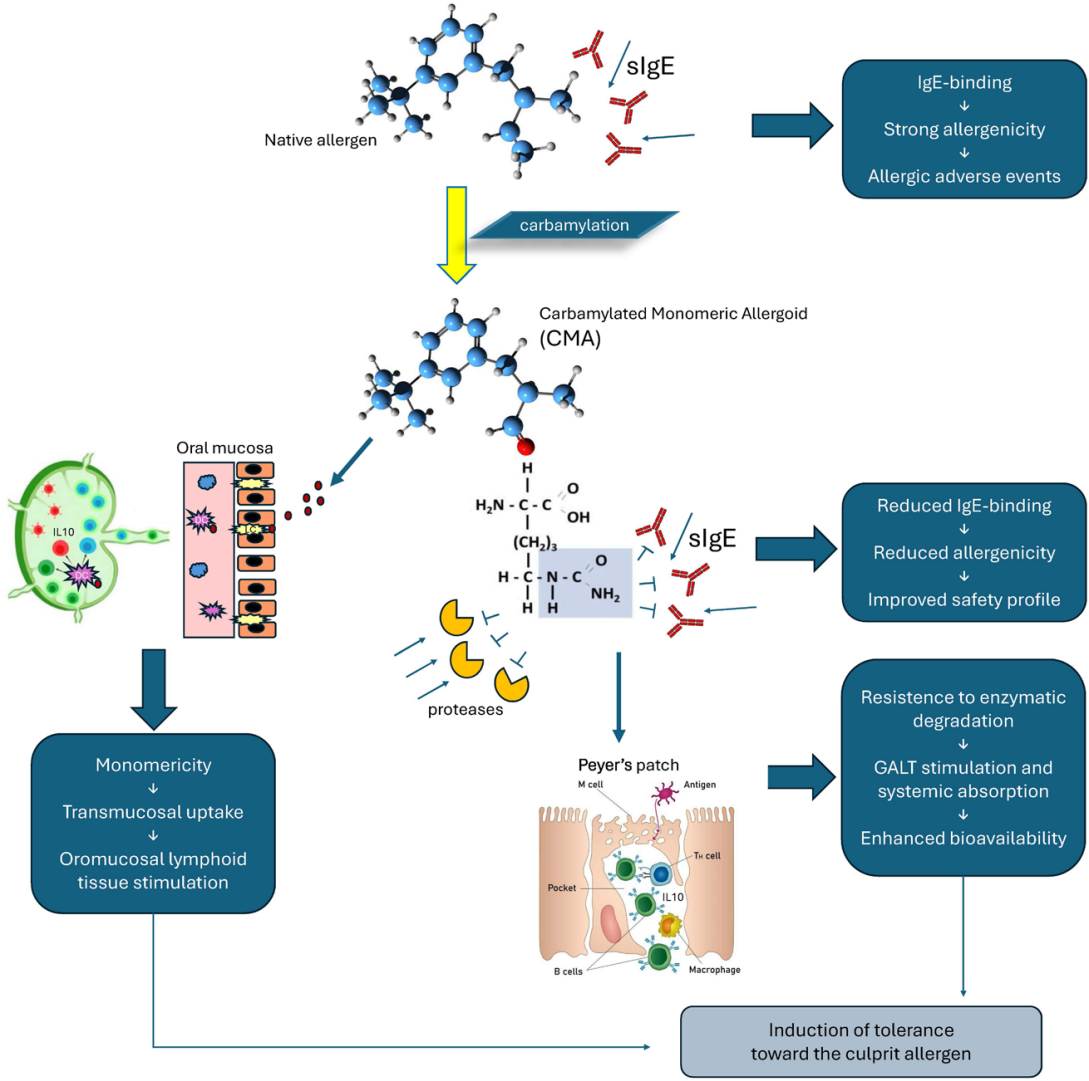
Characteristics of Carbamylated Monomeric Allergoid. DC, dendritic cells; GALT, gut associated lymphoid tissue; IL-10, interleukin 10; LC, Langheran’s cells; sIGE, specific immunoglobulins E.

A peculiar chemical reaction has been specifically developed and patented to transfer the properties of allergoids to sublingual formulations, thus adding reduced allergenicity to the advantages of SLIT (Fig. 1). CMAs are chemically modified allergens synthesized by substituting ε-amino groups of allergen lysine residues (amino acids critical for IgE recognition) using potassium cyanate (KOCN) [39]. This reduces IgE-binding activity (reducing allergenicity) while maintaining immunogenicity. Several postmarketing experiences documented the very limited occurrence of adverse reactions [36]. A recent pharmacovigilance study [40] confirmed these findings with a rate of ADR to monomeric allergoid-based AIT of 0.0004% of the administered doses, which was far lower than the reported rates for native allergen AIT products [41]. When injected in a pediatric population, the CMAs appear safe and well tolerated as highlighted in another pharmacovigilance surveillance [42]. From January 2009 to September 2022, the incidence of ADRs was 0.000077% for injective and 0.000004% for sublingual administrations of CMAs.

Another feature of CMAs is the carbamylation process retains the allergen’s original monomeric nature producing an allergoid with a much smaller molecular weight compared with others (12–40 kDa vs >1,000 kDa). This enables easier absorption through oral mucosa. Carbamylation confers some resistance to allergenic degradation and increases stability when mixed with proteolytic enzymes found in saliva [32]. Moreover, this event seems to favor the oral bioavailability of the extract, as shown in pharmacokinetics studies (Fig. 1) [43].

## 5. Mechanisms of action of CMAs

Mechanistic studies suggest that SLIT shares common pathways with SCIT since the oral mucosa is a site of intense immune activity, and pharmacokinetic studies have described the process of mucosal absorption. One proposed mode of action is that AIT causes a significant decrease in Th2 cytokines and Th2 effector cell activity, increased secretion of anti-inflammatory cytokines such as IL-10 and transforming growth factor-beta (TGF-β), and increased numbers and activity of Treg cells, consequently diminishing the allergic reaction to specific allergens [24]. Even a short course (2 months) with no up-dosing phase of SLIT with monomeric allergoid was shown to decrease allergen-specific proliferation while increasing TGF-β and IL-10 production. The increase in these 2 cytokines suggested increased regulatory T-cell activity [44–46]. More recently, clinically successful HDM SLIT with monomeric allergoid in children treated for 1 year demonstrated repatterning of the differentiation status of Tregs, with high rates of the most suppressive Treg subtypes (activated and effector). This was associated with the generation of cells lacking CD45RA that characterizes memory T-cells with increased activity upon re‐exposure to the antigen, thus suggesting that SLIT also induced Treg inhibitory function, likely compensating for the underrepresentation of Tregs observed in allergic patients. These changes were statistically correlated with observed clinical outcomes: improvement in visual analog scale (VAS) for rhinitis scores and allergic rhinitis and its impact on asthma severity classification, and increased asthma control test scores [47].

The antigens in SLIT tablets are captured and processed by oral APCs within 15 to 30 minutes, which then migrate to cervical lymph nodes within 12 to 24 hours. Interaction of these APCs with naive CD4^+^ T-cells induces suppressive activity from Th1/reg T-cells within 2 to 5 days. The CD4^+^ T-cells then migrate into the blood and tissues, producing a long-term allergen-specific tolerance. The advantage of the sublingual route over other mucosal routes is that the antigen is captured and processed by APCs before any substantial proteolytic degradation occurs, which preserves T and B-cell epitope integrity [48]. Human saliva contains more than 3,000 proteins, including enzymes, hormones, antibodies, and cytokines [49], which may degrade the allergens and reduce their potency. It is therefore important that the tablets breakdown quickly, releasing the allergens to interact with the sublingual mucosa for at least 5 minutes to maximize uptake of the allergens. This enhances the APCs (specifically Langerhans cells) TGF-β and IL-10-producing properties [50].

A small study [43] comparing the pharmacokinetics of 3 SLIT products (allergen solution, allergen tablet, and allergoid tablet) suggested that most of the absorption of the allergens into the blood occurred only after swallowing the compound—no direct absorption through the oral mucosa was detected. It appears the allergens were absorbed instead through the gut mucosa. The authors speculated that part of the allergoid protein could be absorbed through the gastrointestinal tract with little or no degradation. It is known from proteomic studies that carbamylation of proteins at the side chain amino groups of lysines and arginine residues hinders protease digestion. The allergoid’s chemical structure resists enzymatic degradation leading to higher absorption of the allergoid and higher levels in the bloodstream compared with the other 2 products (*P* < 0.05). The allergens from all 3 products persisted in the mouth for hours, which is consistent with the hypothesis that oromucosal immunity is also involved in the action of local AIT.

## 6. Efficacy and safety of CMAs

AIT is largely recognized as the only causal treatment of allergic diseases that targets the underlying pathophysiology and may have a disease-modifying effect in addition to the antisymptomatic effect. Either the SCIT or SLIT routes may be used according to evidence-based clinical recommendations based on several systematic reviews and meta-analyses on the efficacy in subjects with AR and AA. The level of evidence, however, is not the same for allergens of all different natures and for all population targets. Grass pollen and HDM represent the most investigated AIT, with short-term efficacy in adults being the most documented outcome. In general, a product-specific evaluation of evidence is recommended. The real-life efficacy of this treatment may be severely impaired by low patient compliance [51] and perceived substantial delay in the onset of effect after initiation of therapy [2]. However, this limitation may be overcome if personalized routine treatment programs and new media/technological tools are implemented to maintain adherence and persistence. Many allergists recommend at least 2 years of AIT to reap statistically significant and clinically relevant benefits (eg, symptom and IgE decreases) [2, 52], especially in the case of SLIT. To achieve long-term efficacy, it is recommended that a minimum of 3 years of therapy is maintained.

There is insufficient data to determine which is most effective; however, SLIT is generally considered to be less effective than SCIT, or at least slower in effect, because of the difference in administration route (oromucosal vs subcutaneous), resulting in the need for larger doses given over a longer period of time. However, a recent study of SLIT with CMA found statistically significant reductions in symptoms (VAS) and biomarkers (nasal nitric oxide and nasal eosinophils) after only 6 months of treatment in children with AR [53]. This study investigated how to evaluate SLIT effectiveness in the short term considering its effects are rarely observed immediately. Nasal eosinophilia can not only identify good candidates for AIT but also provide a means of measuring treatment efficacy in the short [53] and long term [52].

A systematic review and meta-analysis [32] of earlier studies (1994–2006) investigating CMA tablets for SLIT in pediatric and adult populations found the CMA tablets to be effective and well tolerated. Four double-blind, placebo-controlled studies, 1 open-controlled study, and 1 observational study were used to draw the conclusions. Significant symptom-specific improvements in symptom scores were observed in 3 trials. Typical rhinoconjunctivitis symptoms (eg, rhinorrhea, conjunctivitis, and sneezing) improved in 2 of the studies (*P* < 0.001 and *P* < 0.03), while significant improvements in asthma and rhinitis symptoms were found in the third study (*P* < 0.01). In 3 of the 6 trials, improvement in symptom scores compared with placebo averaged 34%, with the absolute improvement in symptom score from before to after therapy averaged 46% (placebo: 13%). No severe ADRs occurred during any of the trials, only local irritations like itching or swelling of the oral mucosa. These minor side effects did not require treatment discontinuation.

### 6.1. Induction (up-dosing) phase of CMAs

SLIT with CMAs was historically approached by a cautious “updosing” schedule as a legacy of the necessary gradual increase in dosage traditionally used for SCIT, which causes far more side effects and rare severe or even life-threatening events. Initially, the standard induction phase was as long as 14 weeks (traditional schedule) or as short as 16 days (semirush schedule). Subsequent data on the safety of SLIT with CMAs prompted experiments to investigate the feasibility of shorter induction phases (4 days). In one such study [54], 86 young adults with rhinitis and oculo-rhinitis were randomly selected into 1 of 3 parallel groups or controls (no SLIT). Schedule A had no up-dosing so patients started immediately taking 1,000 AU tablets. The other 2 SLIT groups followed a 4-day up-dosing schedule: 500/1,000/1,500/2,000 AU or 300/600/900/1,200 AU. After the up-dosing phase, the maintenance phase was 1,000 AU/wk, taken 2 (HDM) or 5 to 7 (pollen) times per week for the 6-month study period. All patients tolerated the 3 dosage schedules well, with 4 slight side effects recorded in 4 patients during the up-dosing phase and no side effects during the maintenance phase. VAS increased significantly in all 3 SLIT groups compared with controls (*P* < 0.001) but there were no significant differences between the 3 SLIT groups after 6 months. Symptom–medication scores significantly improved in all SLIT groups compared with controls (*P* < 0.02). In the nasal provocation test, all 3 groups improved over the treatment period (*P* < 0.01), while the controls did not. Another study evaluating a 4-day induction phase in 39 patients (aged 6–49) with rhinitis with/without mild asthma also found good safety and efficacy over the 12-month treatment period [55]. The short induction phases (4 days) when using CMAs becoming more commonplace these days is a definite advantage, as patients receive the maximum dose earlier in their treatment, which may translate to earlier clinical benefit.

### 6.2. CMAs in pediatric populations

Two databases (PubMed and Embase) were searched for studies of CMA SLIT in pediatric populations. Specific search terms included “carbamylated monomeric allergoids” and “allergy,” “allergoids” and “sublingual immunotherapy,” and “allergoids” and “children.” Once retrieved, each search result was analyzed for suitability according to the research questions: “How effective and safe is CMA SLIT in children?” and “What is the efficacy and safety of CMA for SLIT compared to native allergens?” All adult studies were excluded except for 4 mixed-age (adolescents and adults) studies that were included in a separate section “CMAs in mixed-age studies.”

A number of studies of SLIT with CMAs in children exploring different clinical, functional, and biological endpoints have been published (Table 1). Caffarelli et al. [56] investigated their use in 44 children with AA and/or AR and/or conjunctivitis (AA = 10; AA+AR = 6; AA+AR+conjunctivitis = 27; AR only = 1) in a double-blind, placebo-controlled, randomized trial. Children taking the monomeric allergoid grass pollen allergens for 3 months prepollen season experienced significant reductions in total and bronchial symptoms; interestingly, nearly all subjects were asthmatic and obtained benefits. Symptom–medication scores were also significantly lower in the active treatment group compared with the placebo group. No systemic or local adverse reactions were noted. Increases in nasal eosinophil cationic protein (ECP) during the pollen season were noted in both groups but the increases were higher in the placebo group. This may indicate a possible role of SLIT in decreasing eosinophil activation [56]. Another double-blind placebo-controlled trial of monomeric allergoid grass pollen was done in adolescents with asthma and/or rhinitis for 3 months prepollen season for 3 consecutive years. Symptom scores were significantly lower in the active treatment group (*P* < 0.01), as well as bronchial symptoms. The same result was found for antihistamines and bronchodilators use. The benefit achieved improved year by year, suggesting the progressive effect of patients treated with SLIT. No adverse events were recorded [57]. In a third placebo-controlled study children allergic to ragweed with AR with or without AA were treated pre-coseasonally [64]. Patients experienced significantly lower nasal and bronchial symptoms during the pollen season and took less rescue medications (bronchodilators, antihistamines, topical and oral steroids). Only one child reported sneezing as a side effect. An increase in the threshold triggering symptoms during nasal challenge was seen in the active group. All these studies emphasize that the tablet form of SLIT has several advantages compared with conventional drops: (1) dosage is well defined, avoiding possible mistakes when taking drops; (2) the tablet requires 1 to 2 minutes of dissolving time in the mouth, assuring time for the allergens to be absorbed through the buccal mucosa; (3) safety.

**Table 1.** Clinical studies with CMA SLIT in pediatric respiratory allergies

Study	Design	Allergen	Age range	Duration	Subjects	Disease	Outcome	CMA product information
Caffarelli et al. [56]	DBPCRCT	Grass pollen	4–14	12 mo	48	AR, AA	Symptoms/drug reduction; seasonal ECP increase block	Lais grass tablet, by Lofarma. Available in: Italy, Spain, Portugal, Austria, Mexico (approved), Ukraine (approved), and Russia (approved).
Bordignon et al. [57]	DBPCRCT	Grass pollen	14 (average)	36 mo	60	AR, AA	Symptoms/drug reduction	Lais grass tablet, by Lofarma. Available in: Italy, Spain, Portugal, Austria, Mexico (approved), Ukraine (approved), and Russia (approved).
Agostinis et al. [58]	Cohort	Multiple pollen allergens	3–18	6–24 mo	433 (equally distributed among products of 3 manufacturers)	AR, AA	Safety	Lais grass, birch, pellitory, grass-birch mix, grass-olive mix, and grass-pellitory drops, by Lofarma. Available in: Italy, Spain, and Portugal.
Agostinis et al. [59]	Open RCT	Grass pollen	4–16	24 mo	40	AR, AA	VAS improvement	Lais grass tablet, by Lofarma. Available in: Italy, Spain, Portugal, Austria, Mexico (approved), Ukraine (approved), and Russia (approved).
La Rosa et al. [60]	Open RCT	HDM	4–16	18 mo	30	AR, AA	Symptoms/drug reduction; NPT response reduction	Lais Dermatophagoides tablet, by Lofarma. Available in: Italy, Spain, Portugal, Austria, Mexico (approved), Ukraine (approved), Russia (approved), Mongolia (approved), and South Korea (approved).
Ippoliti et al. [61]	Cohort	HDM	9–19	6 mo	40	AR, AA	Symptoms, PEF, ECP improvement	Lais Dermatophagoides drops, by Lofarma. Available in: Italy, Spain, and Portugal.
Marogna et al. [62]	Open RCT	HDM	5–17	36 mo	68	AR, AA	Limiting SMS, NCS, EOS, PD20 deterioration on passive smoking	Lais Dermatophagoides tablet, by Lofarma. Available in: Italy, Spain, Portugal, Austria, Mexico (approved), Ukraine (approved), Russia (approved), Mongolia (approved), and South Korea (approved).
Agostinis et al. [63]	Cohort	HDM, grass pollen	3–5	24 mo	36	AR, AA	Safety, improvement in clinical condition	Lais Dermatophagoides and grass drops, by Lofarma. Available in: Italy, Spain, and Portugal.
Mezei et al. [64]	DBPCRCT	Ragweed pollen	6–17	6 mo	30	AR, AA	Symptoms/drug reduction; NPT response reduction	Lais Ragweed tablets, by Lofarma. Available in: Italy, Austria, Germany, Ukraine (approved), and Hungary (approved).
La Grutta et al. [65]	Open RCT	HDM	7–68	12 mo	56	AA, AR	Symptoms/drug reduction Reduced BHR and nasal EOS	Lais Dermatophagoides tablet, by Lofarma. Available in: Italy, Spain, Portugal, Austria, Mexico (approved), Ukraine (approved), Russia (approved), Mongolia (approved), and South Korea (approved).
Leonardi et al. [66]	Open RCT	Olive pollen	11–40	10 wk	33	AR, AA	VAS improvement; drug reduction	Lais Olive tablet, by Lofarma. Available in: Italy, Spain, and Mexico (approved).
Petrarca et al. [47]	Cohort	HDM	6.5–17.4	12 mo	20	AR, AA	VAS improvement; ACT improvement; improvement of disease severity; repatterning of Treg differentiation	Lais Dermatophagoides tablet, by Lofarma. Available in: Italy, Spain, Portugal, Austria, Mexico (approved), Ukraine (approved), Russia (approved), Mongolia (approved), and South Korea (approved).
Parisi et al. [53]	Cohort	HDM	6–14	6 mo	34	AR, AA	VAS improvement; reduction of nNO and nasal EOS	Lais Dermatophagoides tablet, by Lofarma. Available in: Italy, Spain, Portugal, Austria, Mexico (approved), Ukraine (approved), Russia (approved), Mongolia (approved), and South Korea (approved).
Manti et al. [67]	Cohort	HDM, pollen	6–18	3 y	70	AR, AA	Symptoms/drug/disease severity/sleep disturbances reduction; improvements in quality of life and school performance	Lais Dermatophagoides tablet, by Lofarma. Available in: Italy, Spain, Portugal, Austria, Mexico (approved), Ukraine (approved), Russia (approved), Mongolia (approved), and South Korea (approved).

AA, allergic asthma; ACT, asthma control test; AR, allergic rhinitis; BHR, bronchial hyperreactivity; CMA, carbamylated monomeric allergoid; DBPCRCT, double-blind placebo-controlled randomized trial; ECP, eosinophilic cationic protein; EOS, eosinophils (count); FEV_1_, forced expiratory volume in one second; HDM, house dust mites; NCS, nasal corticosteroids; nNO, nasal nitrous oxide; NPT, nasal provocation test; PD20, provocative dose causing a 20% fall in FEV_1_; PEF, peak inspiratory flow; RCT, randomized controlled trial; SLIT, sublingual immunotherapy; SMS, symptoms medications score; VAS, visual analog scale.

A proof of concept study with grass and HDM CMA in drops formulation administered for 1 to 3 years to children of 3 to 5 years of age suggested that this type of SLIT could also be safely administered to very young children [63]. One episode of abdominal pain occurred in 2 children (5% of patients; 0.071 per 1,000 doses) during the maintenance phase. One episode was mild and transient (<30 minute) and one was moderate, requiring a temporary adjustment of the dose. The clinical evaluation by parents reported high, moderate, and slight improvement in 21, 9, and 4 children, respectively, and no change in 2.

A later study by Agostinis et al. [59] investigated the efficacy and safety of SLIT with CMA at much higher dosages (1,000 AU, 5 times per week) without any up-dosing. Forty allergic children (AA+AR = 24; AR only = 16) were treated pre-/coseasonally for 2 years (12 weeks/y: 8 weeks prepollen season, 4 weeks during it). The VAS mean values were greater in the SLIT group than in controls throughout the 2 years (*P* < 0.05), and no systemic or local adverse reactions were found. An advantage of SLIT with CMA highlighted in this study was the lack of a build-up or up-dosing phase, which allows for maximum doses to be used at the very beginning, with the idea that positive treatment benefits will be seen earlier in a patient-friendly schedule. And because of their low IgE-binding activity preventing IgE-mediated allergen presentation by DCs to Th2 cells, monomeric allergoids keep allergenicity low while maintaining immunogenicity resulting in fewer adverse events [63, 68, 69] This avoids the large increases of allergen-specific IgE observed in SLIT with native grass allergens [16, 17]. A randomized multicenter study comparing sublingual CMA with conventional depot SCIT in asthmatic children 4 to 16 years with HDM allergy showed similar efficacy, while SLIT resulted in a higher compliance rate [60]. Interestingly children with mild asthma caused by HDM allergy and not affected by psychological stress showed significantly greater improvement after treatment with sublingual CMA in terms of symptom score, peak expiratory flow, forced expiratory volume in one second, and serum ECP concentration at different time points in respect to stressed children (with increased serum concentration of neuroendocrine parameters) [61]. A prospective, controlled, and randomized study conducted on 56 patients allergic to HDM allocated to sublingual CMA or pharmacotherapy showed after 1 year greater reduction in mean symptom score (*P* < 0.01) and drug consumption (*P* < 0.001) in the SLIT than in the control group. Methacholine PD20 increased only in the SLIT group (*P* < 0.0005). The reduction of nasal eosinophils was statistically greater (*P* < 0.05) only in the SLIT group indicating SLIT reduces the respiratory airways inflammation more than pharmacotherapy [65]. The study by Parisi et al. [53] suggested the same conclusion when reporting symptom improvement associated with a significant reduction of nasal nitrous oxide and nasal eosinophils in cytological evaluation.

The purpose of a prospective, randomized study was to determine whether passive smoking influences the outcome of therapies in pediatric patients with allergic respiratory diseases in children monosensitized to HDM. After 3 years, while children taking continuous cetirizine worsened or did not improve, SLIT prevented the worsening of all clinical parameters [62].

Children are considered ideal candidates for SLIT given the different administration routes (ie, no needles) and reduced allergenicity that leads to a better safety profile. Because most allergic patients are polysensitized, it is important to treat them with multiple allergens during SLIT for the best possible treatment outcomes. However, there was concern over whether administering more than one allergen may increase ADR occurrence. A head-to-head follow-up study was done comparing ADR occurrence in children receiving a single allergen vs those receiving multiple allergens from 3 different manufacturers equally distributed [58]. Four hundred and thirty-three children (3–18 years) were surveyed with 179 receiving a single extract and 254 receiving multiple allergens. A total of 40,169 doses were given (17,143 single and 23,026 multiple). Of the 254 receiving multiple allergens, 165 had rhinoconjunctivitis and AA, while 101 of the 179 receiving a single allergen had rhinoconjunctivitis and AA. The follow-up period was 6 to 24 months, depending on the duration of the prescribed treatment. At the conclusion of the study, there were 102 reported ADRs in the multiple allergen group and 76 in the single allergen group (no significant difference). Notably, 92.6% of all events were mild and self-resolving, with 93.5% being described as local. The most common side effects in both groups were oral itching/burning and throat irritation. These results concur with a similar study done in adults [70]. Although routine mixing of multiple allergens for SLIT is generally not recommended, certain combinations may be considered. For example, multiple pollens were used in a study by Agostinis et al. [58].

A 2024 study [67] (Table 1) analyzed a 3-year course of SLIT in a pediatric population (mean age = 14.3 years) with AR and AA. Significant improvements in symptoms and quality of life (*P* < 0.01), along with significant decreases in disease severity, rescue medication use, and sleep disturbances (*P* < 0.01), were noted. School performance also improved (*P* < 0.01). All 70 patients enrolled were 100% compliant throughout the study, 60 were “very satisfied,” 6 “much satisfied,” and 4 “satisfied” with the treatment. Another 2024 study pooling the safety results from 9 randomized, double-blind, placebo-controlled trials in children aged 4 to 17 years found that SLIT tablets to treat grass, ragweed, and HDM AR and/or conjunctivitis were well tolerated, with 98% of all treatment-related ADRs being mild or moderate and most were localized reactions (e.g., oral pruritus, throat irritation, and ear pruritus) [71]. Finally, an interesting comparative study of 2 SLIT medications, CMAs, and native allergen extracts, was carried out in Korea [72]. Both products significantly improved symptom scores after 1 year of treatment and medication use significantly decreased. ADR incidence was markedly different between the 2 groups, however, with only 6.2% of those taking CMAs vs 33.3% (*P* < 0.05) taking the native allergen extracts.

### 6.3. CMAs in mixed-age studies

The absence of substantial differences in dose–response effect for 2 doses of CMAs in SLIT was found in a study [73] of patients sensitized to HDM including children. Thirty-four patients aged 10 to 52 years were given either 1,000 AU or 2,000 AU for 12 weeks and then were examined for allergen local reactivity assessed by change of the threshold of allergen concentration for a positive nasal provocation test before and after the treatment and change in the mean percentage fall of peak nasal inspiratory flow, symptoms, and adverse reactions. These findings could be explained by the fact that the threshold for efficacy is more easily reached through the enhanced bioavailability of the extract consequent to selective chemical modification [68, 74, 75]. The CMA seems able to partially resist enzymatic digestion after swallowing, which allows for enhanced systemic immunological stimulation through the amount of active principle reaching the gastroenteric lymphoid tissue [75, 76]. Substantial clinical benefits can be derived from the administration of relatively low doses compared with vaccines based on native allergens that are largely degraded in the gastroenteric tract and likely work only through oromucosal uptake.

In a randomized open-label study [77] of 61 children and young adults with a history of AA and AR or rhinoconjunctivitis, HDM monomeric allergoid tablets were administered at either 2,000 or 4,000 AU/wk for 12 months. A significant improvement in symptoms was found in both groups (*P* < 0.001) compared with their baselines but there was no difference between the 2 groups. There was also no difference between the groups in the use of additional antiallergic medications. The provocating dose of methacholine producing a 20% drop in forced expiratory volume in one second (PD20) significantly increased after 12 months in the heavier dose group only. There were no systemic side effects reported in any patients. Again, as in the previous study mentioned [73], CMAs are effective across a wide range of doses. However, Leonardi et al. [77] concluded that because of the risk of AR progressing toward AA, the more significant effect of the higher dosage on bronchial reactivity may justify the use of higher dosages. Conversely, another study comparing 1,000 AU or 3,000 AU of HDM CMA weekly for 1 year found slightly better clinical and immunological results with the highest schedule [78].

A significant decrease in bronchial hyperreactivity to methacholine compared with controls receiving pharmacotherapy was observed in a study of a mixed population with asthma due to grass pollen treated with sublingual CMA. This was associated with a significant improvement in both rhinitis and asthma, parallel to a reduction of drug usage over 3 years, again without relevant side effects [79]. Similarly, SLIT seemed to give some protection against asthma or bronchial hyperreactivity worsening when given to subjects with HDM allergy if pharmacological therapy failed after 12 months [62].

A multicenter prospective, double-blind, randomized, placebo-controlled phase III study evaluated the safety and efficacy of CMA grass in a mixed-age (12–64 years) population, including 14 adolescents with AR with or without asthma [80]. Overall, 98 randomized subjects receiving treatment for 7 to 9 months pre-coseasonally experienced significantly reduced combined symptoms-medication scores compared with placebo during pollen peak days (*P* < 0.0001) and 3 months of grass season (*P* < 0.0001). The proportion of patients experiencing ADRs was similar in the active (26) and placebo groups (20), with no severe local reactions occurring. The authors concluded the grass allergoid tablet was associated with a clinically relevant significant improvement in AR symptoms and use of antiallergic medications and a low incidence of mild ADRs.

## 7. Gap of knowledge and future studies

It is important to note that there is a lack of large-scale phase III studies of CMA SLIT. To confirm the efficacy and long-term safety of CMAs, multicenter multinational studies of CMA SLIT in pediatric populations need to be conducted. AIT with allergoids instead of natural extracts has been shown to be safer in terms of ADR frequency [39, 74] but these are small-scale studies. In addition, comparative studies of CMA vs native extracts are few [72]. To explain this knowledge gap, one must consider that CMAs were first introduced without a formal development plan as requested by recent regulations for new products and were largely used since the early 1990s as a named patient product, gaining real-world evidence from well-established use over more than 30 years.

AIT with natural allergen extracts is a viable disease-modifying treatment for long-term symptom relief in allergic patients, and it can also prevent the progression of AR to asthma [81]. Some data suggest that CMAs may also have this effect [82, 83]. However, natural extracts are more allergenic than CMAs; thus, they have a greater propensity to cause ADRs, including severe ones like anaphylaxis or requiring epinephrine administration [84]. This highlights the need for further large-scale safety and efficacy studies of CMA SLIT, including some comparison studies of CMAs vs native extracts. At this point, we cannot conclude CMA SLIT is comparable or even superior to native allergen SLIT. However, current published data suggest only a limited number of patients with coexisting rhinitis and asthma treated with CMA experience local or systemic ADRs in comparison to conventional SLIT (Table 2) [85]. The mechanisms of CMAs are mainly the induction of cellular tolerance and the restoration of innate immunity. Future studies are needed to further elucidate the specific immunological pathways involved in the therapeutic effects of CMAs [44–47, 86].

**Table 2.** Adverse reactions reported in clinical studies summarized in this review.

	Caffarelli et al. [56]		Bordignon et al. [57]		Agostinis et al. [59]	La Rosa et al. [60]	Ippoliti et al. [61]	Marogna et al. [62]	Agostinis et al. [63]	Mezei et al. [64]		Lombardi et al. [79]	La Grutta et al. [65]	Leonardi et al. [66]	Di Gioacchino et al. [78]	Leonardi et al. [77]	Manti et al. [67]	Petrarca et al. [47]	Jung et al. [72]		Compalati and Frati [80]		Parisi et al. [53]
Condition in CMA group	7 with AA, 1 with AR, 3 with AA+AR, 13 with AA+ARC		With ARC and/or AA		11 with AR+AA, 9 AR	With AA±ARC	28 mild AA, 12 with AA+AR	With rhinitis and asthma	17 with persistent AA, 12 intermittent AA, 33 with ARC	29 with ARC, 11 with AA+ARC		8 with AR, 18 AR+AA	With mild persistent AA±AR	7 with AR, 14 with AA+AR	With persistent moderate or severe AR	2 with AA, 48 with AA+AR, 11 with AA+ARC	64 with AA±AR, 6 with AR	13 with persistent AR, 7 with AA+AR	125 with AR, 10 with AR+AA, 11 with atopy		47 with ARC, 15 with AR, 3 with ARC+AA		With AR
Group	CMA (24)	Placebo (20)	CMA (30)	Placebo (30)	CMA (20)	CMA (30)	CMA (40)	CMA (34)	CMA (36)	CMA (40) (20 pediatric)	Placebo (20) (10 pediatric)	CMA (26)	CMA (33)	CMA (21)	CMA (48)	CMA (61)	CMA (70)	CMA (20)	CMA (146) (115 pediatric)	Staloral (147) (109 pediatric)	CMA (47) (10 pediatric)	Placebo (47) (4 pediatric)	CMA (34)
Number of ADRs (related AEs)	0	0	0	0	0	0	0	NA	2	4	1	NA	0	1	2	0	0	0	9	49	61	61	0
Participants with ADRs, n (%)	0 (0%)	0 (0%)	0 (0%)	0 (0%)	0 (0%)	0 (0%)	0 (0%)	NA	2 (5.5%)	4 (10%)	1 (5%)	NA	0 (0%)	1 (4.7%)	2 (4.2%)	0 (0%)	0 (0%)	0 (0%)	9 (6.2%)	49 (33.3%)	26 (55%)	20 (42.5%)	0 (0%)
Participants with allergic ADRs	0 (0%)	0 (0%)	0 (0%)	0 (0%)	0 (0%)	0 (0%)	0 (0%)	NA	2 (5.5%)	4 (10%)	1 (5%)	NA	0 (0%)	1 (4.7%)	2 (4.2%)	0 (0%)	0 (0%)	0 (0%)	9 (6.2%)	49 (33.3%)	9 (19.1%)	8 (17%)	0 (0%)
Participants discontinued due to ADRs	0 (0%)	0 (0%)	0 (0%)	0 (0%)	0 (0%)	0 (0%)	0 (0%)	NA	0 (0%)	0 (0%)	0 (0%)	0 (0%)	0 (0%)	0 (0%)	0 (0%)	0 (0%)	0 (0%)	0 (0%)	3 (2%)	22 (15%)	1 (2.1%)	1 (2.1%)	0 (0%)
Serious ADRs	0	0	0	0	0	0	0	-	0	0	0	0	0	0	0	0	0	0	0	0	1 (unrelated)	0	0
Local ADRs	0	0	0	0	0	0	0	-	2	0	1	NA	0	NA	2	0	0	0	2	21	5	3	0
Oromucosal	0	0	0	0	0	0	0	-	0	0	0	Unspecified cases	0	-	2	0	0	0		15	4	2	0
Gastrointestinal	0	0	0	0	0	0	0	-	2	0	1	0	0	-	0	0	0	0	2	6	1	1	0
Systemic ADRs	0	0	0	0	0	0	0	-	0	4	0	0	0	NA	0	0	0	0	5	22	15	11	0
Upper respiratory	0	0	0	0	0	0	0	-	0	3	0	0	0	-	0	0	0	0	0	0	5	2	0
Lower respiratory	0	0	0	0	0	0	0	-	0	0	0	0	0	-	0	0	0	0	0	0	2	3	0
Cutaneous	0	0	0	0	0	0	0	-	0	1	0	0	0	-	0	0	0	0	5	22	1	1	0
Aspecific	0	0	0	0	0	0	0	-	0	0	0	0	0	-	0	0	0	0	2	2	7	5	0
Epinephrine administration	0	0	0	0	0	0	0	-	0	0	0	0	0	0	0	0	0	0	0	0	0	0	0

AA, allergic asthma; ADR, adverse drug reaction; AE, adverse event; AR, allergic rhinitis; ARC, allergic rhinoconjunctivitis; CMA, carbamylated monomeric allergoid; NA, not available.

## 8. Conclusions

SLIT is a pathophysiological-altering treatment for allergic disease and has been used efficaciously and safely in many population types. Allergoids were first developed to reduce allergenicity and improve the safety of AIT products. The more recently developed chemical modifications occurring during the carbamylation process have given CMAs several advantages over other SLIT products: small size for easier absorption through the mucosa, greater resistance to proteolytic degradation, greater bioavailability, and reduced allergenicity with full immunogenicity. The tablet form allows for accurate dosing and compliance is easy to monitor. If children are suggested as the best target population for early intervention on the natural progression of allergic disease, recent research data published on HDM CMAs indicate interesting immunological findings and impact on target organ inflammatory markers.

## Conflicts of interest

The authors have no financial conflicts of interest.

## Authors contributions

C-KK: Conceptualization of study and writing of manuscript, review and feedback, and approval of final draft. EC: Writing of manuscript, review and feedback, and approval of final draft. ZC: Conceptualization of study and writing of manuscript, review and feedback, and approval of final draft.
